# Accuracy of Protein-Protein Binding Sites in High-Throughput Template-Based Modeling

**DOI:** 10.1371/journal.pcbi.1000727

**Published:** 2010-04-01

**Authors:** Petras J. Kundrotas, Ilya A. Vakser

**Affiliations:** Center for Bioinformatics and Department of Molecular Biosciences, The University of Kansas, Lawrence, Kansas, United States of America; National Cancer Institute, United States of America and Tel Aviv University, Israel

## Abstract

The accuracy of protein structures, particularly their binding sites, is essential for the success of modeling protein complexes. Computationally inexpensive methodology is required for genome-wide modeling of such structures. For systematic evaluation of potential accuracy in high-throughput modeling of binding sites, a statistical analysis of target-template sequence alignments was performed for a representative set of protein complexes. For most of the complexes, alignments containing all residues of the interface were found. The full interface alignments were obtained even in the case of poor alignments where a relatively small part of the target sequence (as low as 40%) aligned to the template sequence, with a low overall alignment identity (<30%). Although such poor overall alignments might be considered inadequate for modeling of whole proteins, the alignment of the interfaces was strong enough for docking. In the set of homology models built on these alignments, one third of those ranked 1 by a simple sequence identity criteria had RMSD<5 Å, the accuracy suitable for low-resolution template free docking. Such models corresponded to multi-domain target proteins, whereas for single-domain proteins the best models had 5 Å<RMSD<10 Å, the accuracy suitable for less sensitive structure-alignment methods. Overall, ∼50% of complexes with the interfaces modeled by high-throughput techniques had accuracy suitable for meaningful docking experiments. This percentage will grow with the increasing availability of co-crystallized protein-protein complexes.

## Author Summary

Protein-protein interactions play a central role in life processes at the molecular level. The structural information on these interactions is essential for our understanding of these processes and our ability to design drugs to cure diseases. Limitations of experimental techniques to determine the structure of protein-protein complexes leave the vast majority of these complexes to be determined by computational modeling. The modeling is also important for revealing the mechanisms of the complex formation. The 3D modeling of protein complexes (protein docking) relies on the structure of the individual proteins for the prediction of their assembly. Thus the structural accuracy of the individual proteins, which often are models themselves, is critical for the docking. For the docking purposes, the accuracy of the binding sites is obviously essential, whereas the accuracy of the non-binding regions is less critical. In our study, we systematically analyze the accuracy of the binding sites in protein models produced by high-throughput techniques suitable for large-scale (e.g., genome-wide) studies. The results indicate that this accuracy is adequate for the low- to medium-resolution docking of a significant part of known protein-protein complexes.

## Introduction

Protein interactions are a central component of life processes. The structural characterization of these interactions is essential for our ability to understand these processes and to utilize this knowledge in biology and medicine. Experimental approaches, primarily X-ray crystallography, are producing an increasing number of protein structures (www.pdb.org), which to a certain extent are representative of a significant part of the “protein universe.” However, the overall number of proteins by far exceeds the capabilities of the experimental structure-determination approaches [1],[2]. The answer to this discrepancy is computational modeling of protein structures. The modeling not only can supply the vast majority of protein structures, but also, importantly, is indispensable for understanding the fundamental principles of protein structure and function.

Computational structure prediction methodology historically started with *ab initio* approaches based on approximation of fundamental physical principles, and continues to develop in this direction for the goal of learning the principles of protein structure and function. However, for the purpose of predicting protein structures, it has largely evolved to comparative techniques based on experimentally determined structural templates (to a significant extent due to the increasing availability of such templates). Such approaches are faster, more reliable, and provide accuracy increasingly comparable with experimental approaches [3].

A similar trend is underway in structural modeling of protein interactions - protein docking [4],[5]. Because of the nature of the problem, the *ab initio* structure-based methods in docking (prediction of the complex from known separate structures) are relatively more reliable than those in individual protein modeling (docking rigid-body approximation has only six degrees of freedom and has an established record of practical applications). However, the knowledge-based docking approaches, including the template based ones, are rapidly developing, following the increasing availability of the experimentally determined structures of protein-protein complexes, which generally are more difficult to determine than the structures of individual proteins [6]–[8]. It was established by studies based on different sets of proteins that proteins similar in sequence, fold and/or function share similar binding sites [9]–[12]. Quantitative guidelines for quality of homology modeling of protein complexes were provided by Aloy and others [13] where it was demonstrated that sequence identities >40% yield high similarity of protein-protein binding sites.

The modeling techniques for proteins and protein complexes applicable to entire genomes have to be high-throughput by design. This reason, along with the still limited availability of templates, causes the modeling techniques to combine high-resolution approaches, when available and computationally feasible, with low-resolution capabilities, for broad coverage of the proteome/interactome. Such low-resolution approaches still are capable of predicting essential structural characteristics of proteins and protein interactions, including the binding sites [14]–[16], macromolecular assemblies [17] and binding modes for protein-protein [18],[19] and protein-ligand [20] complexes.

For template based docking (based on co-crystallized protein-protein templates), the degree of similarity to the templates is key to the accuracy of the docking. For *ab initio*, as well as some knowledge/template based docking techniques, the accuracy of the resulting structures is directly dependent on the accuracy of the individual participating proteins, which in its turn is based on the similarity to the templates of individual proteins. In both cases, the critical component affecting the docking outcome is the ability to model the structures of the binding sites. Although one can argue that the structure of the whole proteins is important in general, the binding sites are the parts that have a direct effect on the accuracy of the predicted complex. Earlier estimates showed that the binding site accuracy of ∼6 Å C^α^ RMSD is sufficient for low-resolution *ab initio* docking [19] (<3 Å C^α^ RMSD for small ligand-receptor docking [20]), with even lower accuracy suitable for meaningful docking prediction by template based docking (Sinha et al. in preparation).

In the current study we present a systematic analysis of the sequence alignment and subsequent modeling accuracy of known protein-protein binding sites. The analysis is performed and validated on the Dockground comprehensive dataset of co-crystallized protein-protein complexes [21]. According to the purpose of this study (the assessment of high-throughput modeling capabilities for genome-size systems) the modeling was deliberately performed in a high-throughput fashion using standard alignment (BLASTPGP [22]) and comparative modeling (NEST [23]) programs, as opposed to more detailed and sophisticated (but also more computationally expensive) multi-template procedures. The results show that for a significant part of the proteins the binding sites can be modeled with accuracy that would ensure meaningful docking, even in cases of alignments considered poor for modeling of monomeric proteins. Thus, structural modeling of protein-protein interactions can often be performed by means simpler than those typically used for modeling of monomeric proteins, despite the fact that protein-protein interactions in general are on the next complexity level relative to individual proteins. However, further advancement of large scale, high-throughput docking requires progress in experimental determination of structural templates.

## Results/Discussion

### Interface Coverage in Local Alignments

To assess the potential quality of binding site modeling, the sequences of 658 two-chain complexes (Table 1) were subjected to PSI-BLAST search for homologous sequences in the PDB data bank. The following alignments were excluded from the resulting pool: (a) statistically insignificant alignments with expectation value *e*>1 and (b) alignments with target/template difference <10 residues. The latter allowed us to avoid a bias in alignment statistics caused by overrepresentation of certain groups of the proteins and their mutants in PDB. The resulting 66,706 alignments were further analyzed in terms of the target sequence coverage *q* (see Methods, Eq. 1), and coverage of the target interface residues *q_int_* (Eq. 2), with an emphasis on alignments with *q_int_* = 100% (hereafter referred to as full interface coverage, or FIC, alignments). A residue of the target complex was assigned to the interface if the distance between any atom of the residue and any atom of the other subunit in the complex was less than the sum of the van der Waals radii of the atoms plus the diameter of water molecule 2.8 Å. An alignment was considered FIC with a level of tolerance that allowed one target interface residue to be missing in the alignment. The analysis showed that 37,062 alignments, or 56.1% of the entire alignment pool, are FIC alignments. On the other hand, FIC alignments were observed for both monomers in alignments of 218 target complexes and for one of the monomers in additional 101 targets, which together constitute most (97%) of the dataset.

**Table 1:** Interacting chains with known structure used in calculations.

1acbEI	1e96AB	1h2sAB	1kxqAH	1otsAC	1t9gDS	1x3wAB	2ayoAB
1agrAE	1eaiBD	1h4lAD	1kz7AB	1oxbAB	1ta3BA	1x86AB	2b3tBA
1aroPL	1ebdBC	1h59AB	1kzyCA	1oyvAI	1tafAB	1xb2AB	2b59AB
1avaAC	1eerBA	1h6kAX	1l4dAB	1oyvBI	1tdqAB	1xd3AB	2b5iBA
1avgHI	1efnAB	1h9hEI	1l6xAB	1p5vAB	1te1AB	1xdkBA	2b5iCA
1avwAB	1ewyAC	1he1AC	1l7vAC	1p8vAC	1th1AC	1xdtTR	2bcjAQ
1axiBA	1f02IT	1he8AB	1ldjAB	1p9mCB	1th8AB	1xg2AB	2bfxAD
1ay7AB	1f34AB	1hl6BA	1lfdBA	1p9mAB	1tmqAB	1xk4AC	2bh1AX
1b0nAB	1f3vBA	1hx1AB	1lpbBA	1pk1AB	1tnrAR	1xl3AC	2bkhAB
1b34AB	1f5qAB	1i1rAB	1ltxAR	1ppfEI	1tocBR	1xouBA	2bkkAB
1b6cAB	1f60AB	1i2mBA	1m1eAB	1pqzAB	1tt5AB	1xqsAC	2bkrAB
1blxAB	1f6fBA	1i7wAB	1m27AC	1pvhAB	1tueAB	1xtgAB	2bo9AB
1bmlCA	1f6mAC	1i8lAC	1m2vBA	1pxvAC	1tx4AB	1xu1AR	2bseAE
1bndAB	1f93BE	1iarBA	1m9fAD	1qa9AB	1tx6AI	1y4hAC	2btfAP
1buhAB	1fbvAC	1ib1AE	1ma9AB	1qavBA	1txqAB	1y64AB	2c1mAB
1buiAC	1fccAC	1ibrBA	1mbxAC	1qbkBC	1tygAB	1y8xAB	2c5dAC
1bvnPT	1fleEI	1iraYX	1moxAC	1qo3AC	1u0sYA	1ycsAB	2ckhAB
1bzqAL	1fm9AD	1itbBA	1mq8AB	1r0rEI	1u7fAB	1yvbAI	2ey4AE
1c1yAB	1foeAB	1ixsBA	1mvfAE	1r1kAD	1uadAC	1z0jAB	2ey4AC
1c4zAD	1fqjAB	1j2jAB	1mzwAB	1r4aAE	1ueaAB	1z2cBA	2f9dAP
1c9pAB	1fqjCA	1jatAB	1n0wAB	1r8sAE	1ughEI	1z3eAB	2fi4EI
1cd9BA	1fr2BA	1jdhAB	1nexBA	1rp3AB	1ujwAB	1z3gHA	2g45AB
1choEI	1fs1BA	1jiwPI	1nf3AC	1s1qAB	1ukvGY	1z5yED	2gooAC
1clvAI	1fyhAB	1jk9BA	1nmuAB	1s3sBH	1ul1XA	1z92AB	2gy7AB
1cseEI	1g3nAB	1jmaAB	1npeAB	1s4yBA	1us7AB	1zbdAB	2hppHP
1cxzAB	1g3nAC	1jowBA	1nqlAB	1s6vAB	1usuAB	1zbxAB	2mtaCA
1d2zBA	1g4uSR	1jtdAB	1nt2BA	1sbbBC	1uuzAD	1zc3AD	2sniEI
1d3bAB	1g6vAK	1jtgAB	1nunBA	1sgfGB	1uw4BA	1zlhAB	2trcBP
1d4xAG	1g73AC	1jtpAL	1nvuSQ	1sgpEI	1uzxAB	1zm2AB	3fapAB
1d6rAI	1gc1GC	1jw9BD	1nw9BA	1shwAB	1v5iAB	2a19BA	3hhrCA
1devAB	1gcqAC	1k5dAB	1o6sAB	1shyBA	1v74AB	2a41AC	3proAC
1df9AC	1gh6BA	1k8rAB	1o94AC	1shzAC	1vetAB	2a42AB	3sicEI
1dfjEI	1ghqAB	1k90AD	1oc0AB	1sppAB	1vg0AB	2a5dBA	3ygsCP
1dhkAB	1gl0EI	1kacAB	1oeyJA	1sq0AB	1w1iAF	2a5tAB	4htcHI
1dkfBA	1gl1AI	1kg0BC	1ofhAG	1sq2LN	1w98AB	2a5yBA	4sgbEI
1dkgDA	1gl4AB	1kgyAE	1ofuAX	1stfEI	1wmhAB	2a78BA	
1dmlAB	1glbFG	1ki1BA	1ohzAB	1sv0AC	1wmiAB	2ajfAE	
1dn1AB	1go4AG	1kpsAB	1ol5AB	1svxBA	1wpxAB	2apoAB	
1dowAB	1gpwAB	1kshAB	1oo0AB	1syxAB	1wq1RG	2assBA	
1ds6AB	1gvnBA	1ktkEA	1ophAB	1t0fAC	1wr6AE	2assBC	
1dtdAB	1gxdAC	1ktzBA	1or7AC	1t6bXY	1wrdAB	2auhAB	
1e44BA	1gzsAB	1ku6AB	1oryAB	1t6gAC	1wywAB	2aw2AB	

First four symbols are the PDB code followed by the IDs of interacting chains as in the PDB file.

In the distribution of FIC alignments for different functional classes of proteins (Table 2), notably, but not surprisingly, antibody-antigen complexes representing a fraction (3.6%) of the protein set, produce a significant part of all alignments (17.5%, or ∼970 alignments per target complex), with FIC alignments for both monomers in all 12 cases. Interestingly, in two other functional classes (enzyme-inhibitor and cytokine receptor) the FIC alignments were observed at least for one monomer in almost 100% of cases as well, with the only exception of 1e44, for which PSI-BLAST did not find any homologous sequences in PDB. Out of 11 cases in the ‘other’ functional class, for which no FIC alignments were found, 8 cases had no statistically significant alignments. In 3 complexes (1o6s, 1tt5, and 1zm2) the interface consisted of terminal residues only. Thus the interface coverage could have been significantly reduced by absence of these terminal residues in an alignment, which is often the case in local alignments.

**Table 2:** Number of structures with full interface coverage alignments, *N*_FIC_, for different types of complexes.

Complex type	Total number of structures	Total number of BLAST alignments	*N*_FIC_		
			both monomers	one monomer	none of the monomers
All	329	66706	218	99	12
Antibody-antigen	12	11657	12	0	0
Enzyme-inhibitor	63	9441	42	20	1
Cytokine	25	5183	19	6	0
Other	229	40425	145	73	11

**Figure 1:**
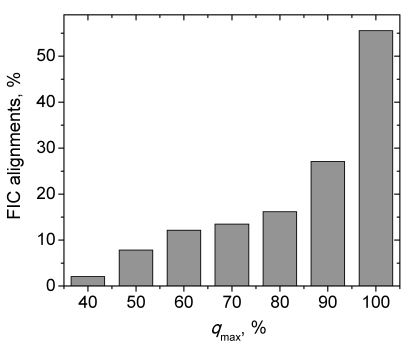
Percentage of alignments with full interface coverage (FIC alignments) in alignment pool produced by PSI-BLAST on the representative set of 329 two-chain complexes at various maximum target sequence coverage *q*_max_.

For further analysis we introduced parameter *q_max_*, the maximal target sequence coverage in a subgroup of alignments and counted the number of alignments (all or FIC only) in subgroups corresponding to *q*≤*q_max_* = 40, 50, 60, 70, 80, 90, and 100% (the entire alignment pool). The results in Figure 1 show that even when the target sequence coverage does not exceed 40%, there is a significant number of FIC alignments (191 out of 9,358 alignments with *q_max_* = 40%). Although these FIC alignments constitute ∼2% of alignments with *q_max_* = 40%, they are still sufficient for statistical analysis. The absolute lengths of these alignments range from 32 to 220 residues (for 86 and 631 residue proteins, respectively), covering from 8 to 40 interfacial residues. The quality of the alignments is rather poor (the range of the expectation values is from 2×10^−48^ to 1.0, the sequence identities vary from 6.5% to 39%, and the gaps constitute up to 32% of the alignments). Such short alignments are generally considered poor in homology modeling of monomeric proteins. However, they can arguably be used for accurate modeling of protein-protein interfaces if all residues of the target interface are present in the alignment. Such interface modeling would provide accuracy sufficient not only for a meaningful analysis of binding properties, but also for docking of 3D models of monomers. Such docking is important for large-scale modeling of protein-protein complexes because modeling based on homology to co-crystallized protein-protein complexes accounts for only 15–20% of all known interactions [24],[25].

### Identity and Similarity of Interface Alignments

**Figure 2:**
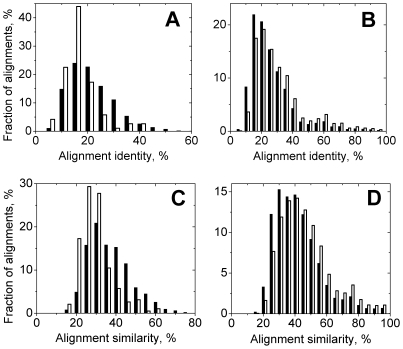
Comparison of distributions of alignment identities and similarities between alignments containing all interface residues and all alignments. The distributions of alignments containing all interface residues are shown by open bars and those of all alignments are shown by closed bars. Panels A and C show distributions for the alignments with maximum query sequence coverage 40% and panels B and D show the distributions for the whole alignment pool irrespectively of query sequence coverage.

It is important to determine if FIC alignments have properties that distinguish them from the whole pool of alignments. The knowledge of such properties would help in “real” homology modeling where interface residues are not known in advance and only the information related to the alignment properties, such as alignment expectation value *e*, and/or alignment identity *a*_iden_ and similarity *a*_sim_ (Eq. 3), is available. For this purpose we compared the distributions of *e*, *a*_iden_ and *a_sim_* for FIC alignments and for all alignments with maximum target sequence coverage *q_max_* (see Figure 2). The results show that *e-*distributions (data not shown) do not differ significantly between the FIC alignments and all alignments, irrespective of *q*_max_ values with a weak tendency of the FIC alignments to have *e* values lower than those in the whole pool of alignments. This difference is small and can be hardly used in practical discrimination of the FIC alignments.

The pattern of distributions of other alignment parameters is different (Figure 2). Whereas for the alignments with *q_max_* = 100% there is no large difference between the FIC and all alignments (Figure 2B, D), the FIC alignments with *q_max_* = 40% show a distinguishable difference from all alignments (Figure 2A, C). For example, the part of the FIC alignments with *a_iden_* between 15 and 20% (84 out of 191) is two times larger than for all alignments (2124 out of 9358; Figure 2A). This difference is even more pronounced for the *a_sim_* distributions (Figure 2C), where the part of alignments with *a_sim_* between 15 and 20% is four times larger for the FIC alignments (33 out of 191 as opposed to 459 out of 9358 for all alignments). We can hypothesize that this is due to a larger evolutionary distance between the target and the template proteins in alignments containing only a small part of the target sequence. Binding sites tend to be more conserved than the rest of the surface in evolutionary related proteins [26]. Such proteins usually correspond to “good” alignments with high target sequence coverage and alignment identity. This assumption is indirectly supported by the distributions of all alignments shown in Figure 2B, D where the fraction of the FIC alignments is larger at higher values of alignment identities and similarities, whereas at lower *a_iden_* and *a_sim_* the situation is opposite.

**Figure 3:**
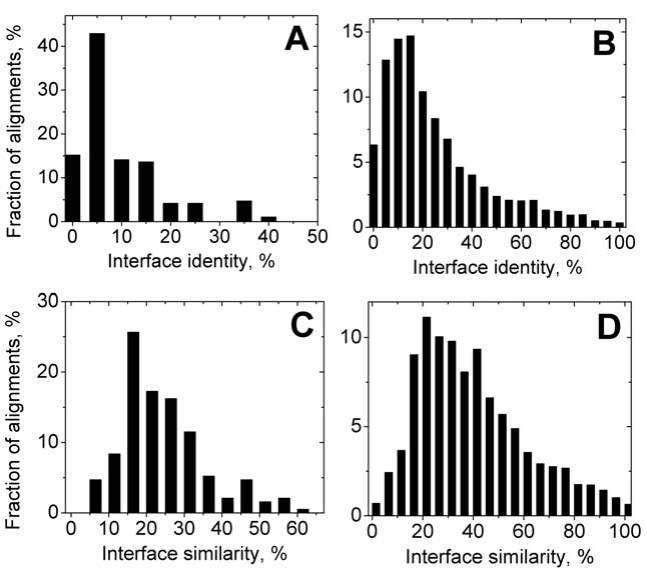
Distributions of interface identities and similarities in alignments containing all interface residues. Panels A and C show the distributions for the alignments with maximum query sequence coverage 40% and panels B and D show the distributions for the alignments irrespectively of query sequence coverage. For the definitions of interface identity and similarity see text.

Figure 3 shows the distributions, similar to those in Figure 2, but only for the residues that belong to the target binding site (these residues do not necessary form continuous stretches of the protein sequence). To avoid ambiguities in definition of interface identity and similarity (Eq. 4) for the alignments with no or little interface coverage, only FIC alignments are considered. The distributions of interface identity *i_iden_* and similarity *i_sim_* qualitatively are similar to distributions of *a_iden_* and *a_sim_*. The main difference is the positions of distribution maxima, which are shifted towards smaller values, compared to corresponding maxima positions in the *a_iden_* and *a_sim_* distributions. The largest difference is in the *i_iden_* distribution for the short alignments, with the maximum for *i_iden_* between 5 and 10% as opposed to 15 to 20% for the *a_iden_* distribution. The distributions for the interface residues are also slightly broader than corresponding distributions for the whole alignments. For example, the peak in *a_iden_* accounts for ∼20% of the alignments while corresponding peak in the *i_iden_* distribution amounts only to ∼15% of the alignments. This is consistent with the previous assumption that alignments with small target sequence coverage are observed for evolutionary distant proteins where interface conservation is not evident. It is important to note that there are significant parts of the alignments with no identity in binding site residues (∼6% for the whole pool of FIC alignments in Figure 3B, and ∼15% for the short FIC alignments in Figure 3A) whereas there are no alignments with zero alignment identity overall (Figures 2A, B). This result by itself is not surprising since alignments with no identical aligned residues have expectation value so high that they are considered statistically insignificant and are not included in the PSI-BLAST output. On the other hand, there are no alignments with zero similarity (no similar residues at all) for the short alignments (Figure 3C) and almost no such alignments (<1%) for the whole alignment pool (Figure 3D). This suggests that even for proteins distant in evolution the interface conservation may play some role, although at more complex level than simple amino acid preservation.

### Probability to Find All Interface Residues in an Alignment

**Figure 4:**
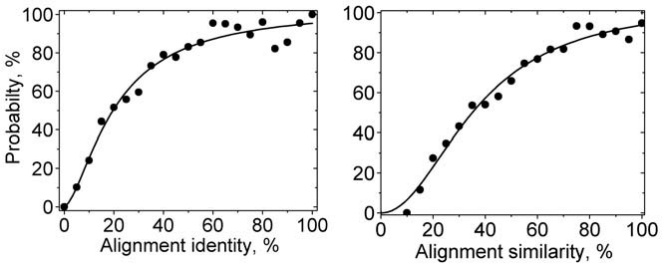
Probability of finding all interface residues inside an alignment as a function of alignment identity and similarity. Curves are least-square polynomial fits to the data points obtained from the analysis of PSI-BLAST alignments for the representative set of 329 complexes used in the study.

For practical modeling of protein complexes it is important to estimate if the interface residues are inside an alignment based on the alignment properties only. For this purpose we determined the number of FIC alignments having certain range of alignment identities/similarities (with a window of 5%) and the number of all alignments having the same range of identities/similarities values. The ratio of those two numbers gives a probability to find all interface residues inside an alignment (or FIC alignment probability) with given identity/similarity. The calculations performed for the alignments with *q*_max_ ranging from 40% to 100% did not find significant differences in the resulting trends. For better visualization (lower statistical noise) Figure 4 shows the FIC alignment probability as a function of alignment identity and similarity for the whole alignment pool (*q*_max_ = 100%) only. Because of representative nature of our dataset of complexes, we can argue that the observed trends in this dataset will hold in the general case. Thus, we can assume that for the alignments with identity >40% (similarity >60%), the probability to find all interface residues in a given alignment is ≥80%. This observation relates to the above suggestion that in the alignments with higher identity/similarity, proteins are closely evolutionary related. It was demonstrated in previous studies of ion binding proteins [27], mitochondrial carriers [28], glycolitic enzymes [29], cyclic dependent kinases [30], and other protein families [26],[31] that the binding sites in closely related proteins are more conserved than the rest of the surface. Thus, the alignment programs (such as PSI-BLAST used in this study) more reliably identify these highly conserved regions, increasing chances to have full binding sites inside an alignment irrespectively of the alignment length. One can argue that this is a nonessential observation since it is well established in homology modeling of individual proteins that model building from the alignment with identity >40% is a trivial task since the fraction of correctly aligned residues in such alignments is approaching 100% (e.g., see Fig. 1B in Ref. [32]). However, the importance of our finding is that it provides a simple recipe for evaluating suitability of a particular alignment for building *partial* homology model of a protein complex of interest with good accuracy in the interface region.

### Partial Structural Models

As mentioned above, there is a significant amount of alignments with low target sequence coverage containing all residues belonging to the interface of the target complex. To assess if such short alignments are useful for structural modeling of protein complexes, we built the structural models and estimated their quality in terms of interface RMSD between the model and the native structures (see Methods) for all FIC alignments with a certain maximum target sequence coverage *q*_max_. To avoid ambiguities caused by possible absence of parts or even all of the interface residues in partial models, the study is restricted to FIC alignments and RMSD of the binding sites atoms. Also we focused on the extreme case of *q_max_* = 40%, although modeling was performed for the alignments with *q_max_* = 50% and 60% as well, with results being qualitatively similar to those for the *q_max_* = 40%. Among the alignments considered, there were no cases for direct homology modeling where sequences of monomers in the target complex are aligned with the sequences from a template complex. The identities of aligned sequence parts in the alignments used to build the models in all cases were well below 40%, which puts them in the “twilight” zone of homology modeling of protein complexes [13].

There were 191 FIC alignments with *q_max_* = 40% for 26 target sequences, among which two were from antibody-antigen complexes, three from enzyme-inhibitor complexes, and the rest from the “other” functional group. This distribution shows no overrepresentation of functional groups compared to the entire dataset. Models were built for all 191 alignments. However, for further analysis we chose a single model per target sequence, based on the highest identity of aligned sequence parts (top model). The results are presented in Table 3. For seven target complexes (∼27%) the top model had interface RMSD<5 Å, which is in line with the estimates of the binding site accuracy needed for meaningful docking predictions [19]. For five complexes, interface RMSD was between 5 Å and 10 Å, which according to the estimates of the docking funnel size [33], can produce near-native matches. Thus we define them as acceptable accuracy models of the monomers (not to be confused with the acceptable accuracy models of the complexes in the CAPRI evaluation http://www.ebi.ac.uk/msd-srv/capri). The FIC alignments were detected in 50% of the complexes with overall alignments considered unsuitable for homology modeling of monomeric proteins. Interestingly, the expectation value of the alignment does not appear to be an appropriate parameter to assess the quality of the resulting model, since in all cases the alignment for the best model did not have the lowest *e*-value among FIC alignments, although the lowest *e*-value observed for the top models alignments was 10^−47^ (1gxd, chain A). For 17 target sequences, the top model was found to be also the best model, *i.e.* model with the lowest interface RMSD. Among 9 cases with different top and best models, only in two cases interface RMSD values were significantly different (the top and the best models in different quality categories; data shown in Table 3 in bold).

**Table 3:** Parameters of the top models produced on the basis of alignments with maximum 40% target sequence coverage and full interface coverage.

Target			Template			Log e(4)	q, %(5)	q_dom_, % (6)	Alignment(7)		Interface(8)		Interface RMSD, Å(9)
PDB and chain ID(1)	Source organism(2)	Biological function(3)	PDB and chain ID(1)	Source organism(2)	Biological function(3)				identity	similarity	identity	similarity	
1avgH	Cow (M)	Blood coagulation	1p3cA*	*B.intermedius* (B)	Proteolysis	−5.16	35.9	–	17.2	30.1	5.3	10.5	7.9
1fqjA	Rat (M)	Detection of light	2g77B	Mouse (M)	GTP-binding	−0.23	34.4	56.6	18.0	26.5	15.8	21.1	3.7
1g4uS	*S.typhimurium* (B)	Dephosphorylation	1he1A	P.aeruginosa (B)	GTPase	−19.00	31.6	90.3	26.5	42.2	34.5	44.8	1.5
1gc1C	Human (M)	T-cell receptor	2z35A	Mouse (M)	T-cell receptor	−0.75	39.5	–	20.3	33.8	12.5	29.2	10.4
1gxdA	Human (M)	Ca, Zn binding	1pex0*	Human	Ca, Zn binding	−47.70	32.3	100.0	34.3	52.0	17.5	45.0	2.2
1h2sA	*N.pharaonis* (A)	Ion transport	1bctA*	*H.salinarium* (A)	H ion transport	−12.05	29.8	–	37.3	58.2	23.8	57.1	19.4
1i8lA	Human (M)	T-cell receptor	1h5bA	Mouse	T-cell receptor	−2.70	39.9	77.4	10.5	29.1	5.6	27.8	7.4
1ixsB	*T.thermophilus* (B)	DNA repair	2ewvA*	*A.aeolicus* (B)	ATP binding	−0.20	31.1	60.0	23.3	39.2	15.0	45.0	22.0
1kg0B	Human (M)	Immune response	1b24A*	*D.mobilis* (A)	Mn ion binding	−0.17	28.7	60.7	33.3	42.6	35.7	35.7	16.9
1kshA	Mouse (M)	GTPase	2it1A*	*P.horikoshii* (A)	ATP binding	−0.60	38.7	–	22.2	36.1	25.0	29.2	31.1
1ktkE	Human (M)	Immune response	1cd8A*	Synthetic construct	MHC-I binding	−0.96	38.5	80.3	17.2	37.4	11.5	26.9	5.6
1m9fD	HIV virus I (V)	RNA binding	1cm5A	*E.coli* (B)	Glucose metabolism	−1.24	33.6	–	38.9	48.2	25.0	25.0	33.4
1mq8A	Human	Leukocyte migration	1t0pB	Human (M)	Mn ion binding	−6.00	28.2	100.0	36.6	51.2	40.0	60.0	1.3
1nexB	Yeast (F)	Protein ubiquitination	1flgA	*P.aeruginosa* (B)	Ca ion binding	−0.66	20.5	–	9.3	33.0	3.2	41.9	53.4
1nt2A	*A.fulgidus* (A)	r,tRNA processing	1sb8A*	*P.aeruginosa* (B)	Coenzyme binding	−0.75	39.1	–	12.4	32.6	6.5	19.4	8.6
1s3sB	Mouse (M)	DNA damage repair	1cz4A*	*T.acidophilum* (A)	ATP binding	−10.40	37.1	–	23.0	40.5	18.5	33.3	4.1
1txqB	Human (M)	Cell division	2bp7A	*P.putida* (B)	Oxidation reduction	−1.52	36.1	–	37.5	59.4	40.0	60.0	21.5
1z3gH	Mouse (M)	-	1fo0A	Mouse (M)	-	−0.43	38.9	73.4	28.9	46.4	35.7	57.1	5.0
2a19A	Yeast (F)	RNA binding	1sljA*	*E.coli* (B)	RNA binding	−4.70	38.9	68.3	15.2	38.0	11.8	29.4	3.2
2assB	Human (M)	Cell proliferation	1nexB	Yeast (F)	Cytokinesis	−2.70	20.8	–	25.4	46.5	36.1	52.8	6.2
2bcjA	Cow(M)	Phosphorylation	1a25A*	Rat (M)	Phosphorylation	−1.68	11.0	–	9.8	31.7	21.1	31.6	48.7
2bcjQ	Rat/Mouse (M)	ADP ribosylation	2g77B	Mouse (M)	GTP binding	−0.55	38.8	65.9	17.6	31.0	14.3	23.8	8.5
**2bcjQ**			1z0aA*	**Human (M)**	**GTP binding**	**−0.48**	**33.4**	**62.1**	**14.4**	**28.0**	**4.8**	**9.5**	**3.8**
2bkkA	*E.faecalis* (B)	ATP binding	2j51A*	Human (M)	ATP binding	−0.77	35.6	–	13.7	22.1	15.0	30.0	32.5
2c5dC	Human (M)	Phosphorylation	1i85A	Human	T-cell receptor	−0.35	36.4	68.9	22.2	32.1	10.4	17.2	18.5
**2c5dC**			**1k8iB**	**Mouse (M)**	**MHC-II binding**	**−2.52**	**39.0**	**73.8**	**14.3**	**29.9**	**3.5**	**13.8**	**4.9**
2gy7B	Human (M)	Phosphorylation	1wgtA	Wheat (P)	Sugar binding	−1.92	37.8	–	11.0	17.2	0.0	5.3	30.8
2mtaC	*P.denitrificans* (B)	Iron binding	1gpeA*	*P.amagasakiense* (F)	FAD binding	−1.23	38.1	–	14.3	26.8	16.7	33.3	22.7

(1) First four symbols are the PDB code followed by ID of the chain as in the PDB file. Asterisk indicates that protein is a monomer in the PDB file.

(2) As provided in PDB file. Letters in parenthesis stand for higher levels of taxonomy classification (V: viruses; A: archaea; B: bacteria; F: fungi; P: plants; M: mammals).

(3) Extracted from PDB GO terms section.

(4) Logarithm of alignment expectation value (*e*-value).

(5) Entire target sequence coverage in the alignment of the model, as defined by equation (1).

(6) Coverage of the target binding domain (for multi-domain structures) in the alignment of the model.

(7) As defined by Eq. 3.

(8) As defined by Eq. 4.

(9) RMSD between C^α^ atoms of the interface residues in the model and the native structure.

For some targets the parameters of the model with the smallest interface RMSD are shown if the best and the top models have substantially different interface RMSD values (in bold).

The data in Table 3 indicate that all FIC alignments for the top models have low sequence and interface identity/similarity, which suggests that target and template proteins in those alignments are evolutionary remote (see discussion in previous sections). Thus, it is interesting to analyze whether there is a preference of target and template proteins in alignments to be from the same organism or from different species. Our analysis suggests no such preference since for good and acceptable models there were 6 target-template pairs from the same organism and 9 pairs from different organisms (corresponding numbers for the wrong models are 5 and 8). This does not support a conclusion from an earlier study [34] that protein-protein interactions are more conserved within one species than across the species. However a statistical analysis on a much larger pool of data is needed to reach a more definite assessment (work currently in progress).

**Figure 5:**
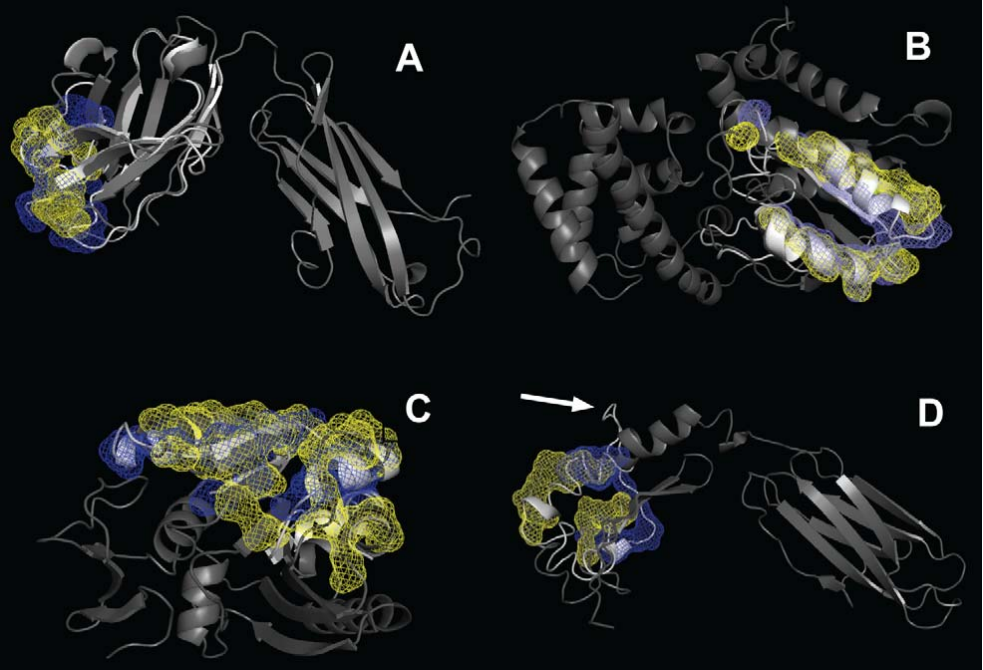
Examples of partial homology models. The models (white ribbons) are superimposed on the target native structures (gray ribbons). (A) Good accuracy model (interface RMSD = 5.0 Å) in the case of target and template proteins from the same organism. Target is malaria transmission blocking antibody 2A8 from mouse, (1z3g, chain H) and template is mouse BM3.3 T-cell receptor α-chain (1fo0, chain A). (B) Good accuracy model (interface RMSD = 3.7 Å) in the case of target and template proteins from different organisms. Target is guanine nucleotide-binding protein alpa-1 subunit from bovine, (1fqj, chain A) and template is yeast RAS-related protein RAB-33 (2g77, chain B). (C) Acceptable accuracy model (interface RMSD = 8.6 Å). Target is fibrillarin-like preRRNA processing protein from *Archaeoglobus fulgidus* (1nt2, chain A) and template is UDP-N-acetylglucosamine 4-epimerase from *Pseudomonas aeruginosa* (1sb8, chain A). (D) Incorrect model (interface RMSD = 16.9 Å). Target is human MHC Class II receptor HLA-DR1 (1kg0, chain B) and template is intron-encoded endonuclease from *Desulfurococcus mobilis* (1b24, chain A). Arrow indicates an incorrect loop which is the cause for large interface RMSD in this model. Blue and yellow meshes indicate positions of the backbone atoms of the interface residues in the model and the native structures, respectively. Other parameters of the models are presented in Table 3.

Figure 5 shows examples of the models, including those for which the target and the template sequences are from the same and from different organisms. One interesting similarity in both cases (Figures 5A and 5B) is that the target proteins have two clearly distinguishable domains and the model structure covers a significant portion of one of the domains, which exclusively participates in the interaction with the other monomer (not shown for clarity). In fact, this feature is common to all good-accuracy models (interface RMSD<5 Å). The data on the binding domain coverage is provided in Table 3 (where applicable). It shows that there is no clear correlation between the binding domain coverage (although it is higher than the entire sequence coverage) and the model quality. Acceptable accuracy models are built for the single domain proteins as well. Figure 5C shows an example of such model. The implication for practical modeling is that if the target protein is predicted to have a domain structure, then it is likely that the accuracy of the homology models produced on the basis of the “bad” alignments will be sufficient to perform a meaningful template-free docking. On the other hand, for homology models of single-domain proteins, methods less sensitive to structural inaccuracies (e.g., structural alignment) should be used. This assessment is supported by a comprehensive study of the template free docking ability to tolerate structural inaccuracies [19], which showed that low-resolution structural features of protein–protein interactions can be determined for a significant percentage of complexes of highly inaccurate protein models (typically up to 6 Å RMSD from the native structure of the monomer). The results were further supported by recent studies of antibody-antigen docking of homology models, which concluded that the homology models yield medium-to-high quality of docking predictions [35]. Further confirmation came in the recent study by Aloy et al. [36] on the structural modeling of yeast interactome where it was found that the use of homology models in docking does not lead to a critical loss of accuracy (assessed by extrapolation of docking results for the unbound X-ray structures).

Our preliminary results on the benchmarking of the template free docking of the modeled structures was performed using GRAMM procedure, according to the goal of this study in the high-throughput fashion that does not involve computationally expensive scoring and structural refinement. The low-resolution criterion for success was: a match with the ligand interface RMSD<8 Å in the top 100 predictions. This RMSD value corresponds to the characteristic size of the binding funnel [33]. Such low-resolution predictions from the coarse-grained global scan are located within the binding funnel and can be further locally refined within the funnel. Higher-resolution docking, and the corresponding more strict success criteria (such as those used in CAPRI), in addition to longer computational times, require higher, non-high-throughput accuracy of the binding site modeling, which is outside the scope of this study. ***The current study is aimed at the models of poor quality that still preserve the acceptable accuracy of the binding site***. According to the above criterion, the success rate for the modeled proteins dropped to 23% from the similarly obtained 43% for the unbound X-ray proteins. However, such success rate is significant for the genome-wide studies. A systematic assessment of docking application to modeled structures of different accuracy is currently in progress.

Table 3 also includes data on the failed modeling (interface RMSD>10 Å). Figure 6D shows an example of such model. The target native structure has the domain structure similar to the successful models described above. The main reason for the incorrect modeling of the interface region is presence of a long stretch of gaps on the template side in the alignment. This is the reason for the incorrect loop (indicated by arrow in Figure 5D), modeled without a template in the vicinity of the interface, which resulted in position shift of the interface residues in the model compared to the native structure (yellow and blue meshes in Figure 5D). Another typical reason for large interface RMSD is the native structure interface having no secondary structure elements (e.g., a loop in enzyme-inhibitor complexes), but the fragment is modeled on a template with distinct secondary structure elements. A large difference between quaternary structures of the native target and the template structures also may lead to large shift of interface residues in the model, even if these residues belong to the same secondary structure elements as in the native structure.

Analysis of organism and functional annotations (Table 3) revealed that both target and template proteins are from the species spanning the entire universe of life - viruses, archaea, bacteria, lower (fungi) and higher (plants and mammals) eukaryotes - and participate in a broad range of biochemical processes. Moreover, there is no clear correlation between source organisms of the target-template pair or the biochemical pathways in which they participate. There are correct models with the target and the template from evolutionary distant organisms (e.g., mammals and archaea), as well as incorrect models with the target and the template from evolutionary close organisms or even the same organism. Similarly, no such correlation was found for the functions of the target and the template proteins, although the functional assignment has limited reliability. This suggests that the current ability to model complexes may not be restricted to certain species and/or functions. However, statistical analysis of a much larger protein interactions dataset, when it becomes available, would be necessary to draw more definite conclusions.

### Concluding Remarks

For systematic evaluation of potential accuracy in high-throughput modeling of binding sites, local sequence alignments were performed in a representative set of protein-protein complexes. The results indicate that for the majority (97%) of the target sequences there is at least one alignment containing all residues belonging to the interface of the target complex (FIC alignments). Significant number of the FIC alignments was observed even when only ∼40% of the target sequence is aligned against the template. The results suggest a simple graphical function for evaluating the probability of finding all interface residues inside a local alignment when only the alignment information is known.

Homology models of the interfaces in target monomers were built based on the FIC alignments with query target sequence coverage <40%. A simple scheme of model ranking based on the alignment identity showed that in ∼50% of cases the structural models have accuracy high enough for protein docking. Alignments that contain only a small portion of the target sequence and have low sequence identity are usually considered poor in modeling of individual proteins. They are used primarily in elaborate and computationally expensive techniques hardly applicable on genome-wide scale. Our results suggest that for the genome-wide structural modeling of protein interactions, simpler and less computationally expensive techniques based on the use of single, local sequence alignment, may yield satisfactory results, given that the interface residues are reliably identified in the alignment. Current methods for predicting protein-protein binding sites based on sequence information alone have limited accuracy (e.g. Refs. [37],[38]). However, because of the on-going significant community efforts in this direction, one may expect emergence of more accurate methods in the near future.

A straightforward template-based modeling of protein complexes is possible on the basis of a co-crystallized template complex. However, previous studies [24],[25] demonstrated that this technique could account only for ∼15–20% of all known interactions, whereas the rest of the protein complexes have to be modeled by other techniques. One possible direction is independent modeling of individual monomers on different templates with further application of docking (either template free or based on structure alignment) to these models. Earlier studies (e.g. Refs [19],[35],[39] and others), as well as the results of this work suggested feasibility of this scenario. However more systematic and comprehensive studies are needed for quantitative guidelines of applicability of the homology models in large-scale structural modeling of protein-protein interactions (study currently in the progress).

## Methods

### Set of Proteins

Hetero-complexes with known 3D structures available in PDB were used in the study. To avoid bias caused by overrepresentation of certain protein families in PDB, we used the representative set of protein complexes from the Dockground resource [21], manually selected and purged at 30% sequence identity level. Out of 523 complexes in the dataset, we further excluded structures with multi-chain interactions and those with large structural defects in the vicinity of the interface, which allowed us to avoid ambiguities in determining binding site residues. The final set consisted of 329 two-chain non-obligate complexes shown in Table 1 (63 enzyme-inhibitor, 12 antibody-antigen, 25 cytokine receptors, and 229 other complexes). This set is based on all protein structures available in PDB; thus the results are not dataset-dependent.

### Software

For 658 sequences in the dataset, the search for sequence homologues was performed by PSI-BLAST [22] implemented in the program BLASTPGP. To broaden the pool of potential templates, the maximum number of hits was set to 2000, with all other parameters set to default values. To obtain the checkpoint file (the position specific scoring matrix PSSM) [22], the search was performed against all sequences in the non-redundant database of sequences (www.ncbi.nlm.nih.gov) with the substitution matrix BLOSUM62 [40] with five iterations. The checkpoint file was used in sequential PSI-BLAST run against all non-redundant sequences in PDB.

The 3D models from the PSI-BLAST sequence alignments were built by program NEST from the JACKAL package developed in Honig's lab [23] using default parameters. Large errors in some template files were repaired by the program PROFIX from the same package. The NEST program was chosen over other popular modeling programs because it yields reliable models fast enough to be used in large-scale calculations (e.g., according to benchmarking of various homology modeling programs [41]) and can be easily incorporated into automatic scripts for generating and updating databases of structural models currently under development in the lab.

### Analysis of Results

Since sequence alignments produced by PSI-BLAST are local by design [22], not all residues of the target sequence are present in the alignment. Thus for the analysis of the alignments we defined the target sequence coverage
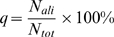
(1)and, similarly, the interface coverage
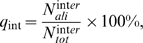
(2)


Where 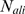 and 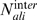 are the numbers of all target residues and the target interface residues, respectively, in the alignment; 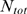 and 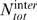 are the total numbers of all residues and the interface residues, correspondingly, in the entire target sequence. We did not analyze whether the template is multi- or monomeric (although the data is available in Table 3) since our goal was to determine the general usefulness of short sequence alignments in binding site modeling, rather than traditional homology modeling of protein complexes where both target and template are multimers. When the target had the multi-domain structure, we also calculated the domain coverage *q_dom_* using formula (1), where *N_ali_* is the total number of the target residues inside the binding domain.

The alignments were further analyzed with respect to the alignment *e*-value as well as their identity and similarity, defined as
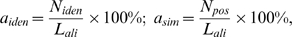
(3)where *L_ali_* is the length of the alignment (number of target residues in an alignment plus gaps in the aligned target sequence), *N_iden_* is the number of aligned identical residue pairs, and *N_pos_* is the number of aligned residues pairs for which substitution matrix displays a positive number (evolutionary favorable substitutions). Similarly, the identity and similarity of the interface residues inside an alignment was defined as
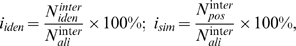
(4)


Where 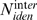 (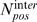) are the number of aligned identical (positive) residue pairs where the residue on the target side belongs to the target complex interface, and 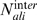 is the total number of the interface target residues in the alignment. To evaluate the quality of the resulting homology model, we calculated the root-mean square distance between C^α^ atoms of the interface residues (interface RMSD), with the native structure of the monomer and its model superimposed by the program TM-align [42]. This measure is different from the RMSD used in the CAPRI evaluation [5], where it is calculated between the interface atoms of the ligand in the native and in the docked matches, after structural superimposition of the receptors. Other widely used modeling quality criteria, such as sensitivity and specificity, are not applicable to our study because they involve true and false-positive/negative predictions that can be defined either for binary predictions of the fact of protein interactions (which is not the case in our study) or in the case of full modeled complex structure with both monomers present.
